# Brief Report: Diabetic Keto-Acidosis (DKA) Induced Hypothermia may be Neuroprotective in Cardiac Arrest

**DOI:** 10.2478/jccm-2023-0004

**Published:** 2023-02-08

**Authors:** Joseph Shiber, Emily Fontane

**Affiliations:** 1 University of Florida College of Medicine, Jacksonville, FL USA

**Keywords:** diabetic ketoacidosis, hypothermia, cardiac arrest, neuroprotection

## Abstract

Despite the decreased survival associated with diabetes with out-of-hospital cardiac arrest and the overall low survival to hospital discharge, we would like to present two cases of OHCA in diabetics who despite prolonged resuscitation efforts had complete neurological recovery likely due to concomitant hypothermia. There is a steady decreasing rate of ROSC with longer durations of CPR so that outcomes are best when <20 minutes compared to prolonged resuscitation efforts (>30-40 minutes). It has been previously recognized that hypothermia prior to cardiac arrest can be neurologically protective even with up to 9 hours of cardiopulmonary resuscitation. Hypothermia has been associated with DKA and although often indicates sepsis with mortality rates of 30-60%, it may indeed be protective if occurring prior to cardiac arrest. The critical factor for neuroprotection may be a slow drop to a temperature <25^0^C prior to OHCA as is achieved in deep hypothermic circulatory arrest for operative procedures of the aortic arch and great vessels. It may be worthwhile continuing aggressive resuscitation efforts even for prolonged periods before attaining ROSC for OHCA in patients found hypothermic from metabolic illnesses as compared to only from environmental exposures (avalanche victims, cold water submersions, etc.) as has been traditionally reported in the medical literature.

## Introduction

Despite the decreased survival associated with diabetes with out-of-hospital cardiac arrest [1] and the overall low survival to hospital discharge [2], we would like to present two cases of OHCA in diabetics who despite prolonged resuscitation efforts had complete neurological recovery likely due to concomitant hypothermia.

## Case Presentation

### Case 1

**Fig. 1:**
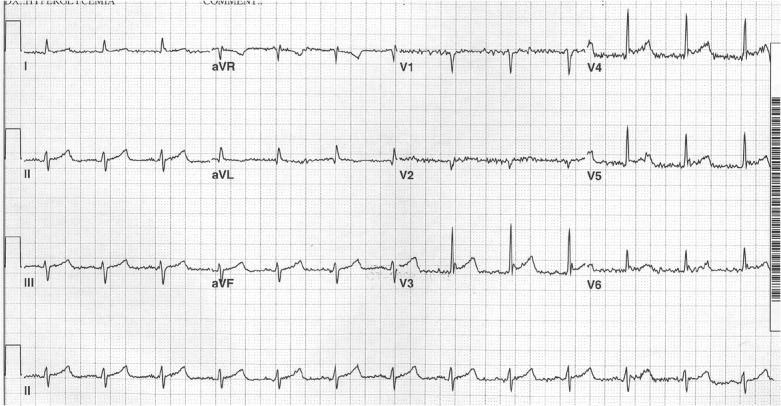
**EKG taken approximately 2 hours s/p ROSC when core temperature now over 30**^0^**C showing Sinus Rhythm with Osborne Waves (elevated J-points) seen in leads V2-3, as well as shivering artifact**.

A 52 year-old woman was found lethargic lying on her bathroom floor after last seen 12 hours earlier when her husband left for work. She had not received any healthcare for the past 10 years until recently being diagnosed with type II diabetes but was not taking any prescribed medications as she was attempting to control her blood sugar using herbal supplements. Her husband reported that she had a 30 pound weight loss over the past two months while complaining of polyuria and polydipsia. On EMS arrival, they found her pulse was 40 BPM, systolic BP was 70 mmHg and glucose >500 mg/dL and she was awake but confused; she went into ventricular fibrillation (VF) as EMS brought her stretcher into the ED. After defibrillation x1 with 200J, she remained in a pulseless wide-complex slow rhythm so CPR was initiated and continued while receiving intravenous epinephrine and crystalloid boluses; Intravenous regular insulin, calcium chloride, and bicarbonate were given for presumed hyperkalemia with DKA. Her trachea was intubated and a right internal jugular intravenous line was placed. Her skin was cold to the touch and her rectal temperature was 22^0^C so an intravenous femoral warming catheter was placed for a rapid goal of 36 ^o^C and warmed blankets were placed over her including wrapping around her head; warmed IV fluids and hydrocortisone 100mg were also given. She received CPR for over 40 minutes before we achieved return of spontaneous circulation (ROSC) as she was now back in sinus bradycardia with heart rate 40-50, and EKG performed 2 hours later demonstrating *Osborne Waves* with shivering artifact (Figure 1). A Dopamine infusion was required to achieve a Mean Arterial Pressure (MAP) of >65 mmHg and was selected specifically for the additional chronotropic effect.

Laboratory studies confirmed DKA with a serum HCO3 of <5 meq/L, Anion Gap of 36, and Glucose of 967 mg/dL. She remained unresponsive on mechanical ventilation and approximately three hours after arrival her core temperature reached 32^0^C so that the intravascular warming/cooling device thermostat was reset to 34^0^C for our goal of post-arrest targeted temperature management (TTM). In the ICU five hours later, she was awake following commands and mouthing words. The TTM was discontinued and her temperature normalized over the next three hours. The next morning she was extubated and transferred to the floor later in the day. She was discharged home 2 days later completely normal neurologically.

## Case 2

A 27 year-old man was found by his mother lying face down unresponsive on his apartment floor. He was diagnosed with type I diabetes at age 11 and had multiple admissions for DKA due to noncompliance with insulin therapy. His past medical history also included asthma, and gastroparesis. She turned him over and began CPR, and he was asystolic when EMS arrived so they transported him with CPR in progress for 10-15 minutes while giving ACLS medications via an intraosseous (IO) line. CPR was continued for approximately 60 additional minutes in the ED after his trachea was intubated and a right femoral venous line was placed. He received intravenous epinephrine every 3-5 minutes (20 mg total), crystalloid boluses, calcium chloride and bicarbonate, as well as defibrillation once for VF before achieving return of spontaneous circulation (ROSC). His rectal temperature was 24^0^C, and glucose was 1,200 mg/dL with a serum HCO3 of <5 meq/L, Anion Gap of 42 and base deficit of -28. An esophageal warming/cooling device was placed set to a temperature of 36^0^C, and an external warm-air circulating blanket was placed over him. An insulin infusion and warmed fluids were administered.

Despite having anuric acute kidney injury (creatinine >5 mg/dL), rhabdomyolysis (CPK >50,000 U/L), shock liver (AST >3,500 U/L), severe ARDS (pO2/FiO2 ratio <100) and acute myocardial stunning (EF 30%) requiring CRRT and epinephrine infusion he was able to write notes using his cellphone 8 hours later when his temperature was now 36^0^C. He was extubated 4 days later and discharged home completely normal neurologically with normal cardiac and hepatic function but still requiring intermittent dialysis for several weeks. He has had numerous repeat admissions for DKA in subsequent years despite endocrinology changing his insulin regimen to a pump, case management involvement, psychiatry evaluation, and intense parental interventions including attempts to gain legal guardianship. Tragically four years after this index presentation, he was found dead at his home at age 31.

## Discussion

There is a steady decreasing rate of ROSC with longer durations of CPR so that outcomes are best when <20 minutes compared to prolonged resuscitation efforts (>30-40 minutes) [3]. It has been previously recognized that hypothermia prior to cardiac arrest can be neurologically protective even with up to 9 hours of cardiopulmonary resuscitation [4]. Hypothermia has been associated with DKA and although often indicates sepsis with mortality rates of 30-60%, it may indeed be protective if occurring prior to cardiac arrest [5]. The critical factor for neuroprotection may be a slow drop to a temperature <25^0^C prior to out of hospital cardiac arrest (OHCA) as is achieved in deep hypothermic circulatory arrest for operative procedures of the aortic arch and great vessels [6]. The cause of hypothermia associated with DKA is presumed to be the lack of insulin preventing the intracellular movement of glucose to be used as a substrate for heat production [7]; other causes of hypothermia in diabetic patients include impairment of heat conservation methods due to autonomic dysfunction failing to induce effective peripheral vasoconstriction, as well as reduced muscle mass producing insufficient heat output [8,9]. Severe hypothermia may indeed also exacerbate the worsening diabetic state by inhibiting the endogenous release of insulin as well as impairing the cellular effect of exogenously administered insulin [6,10]. These factors are the basis for the imperative to rapidly warm a DKA patient to >30-32^0^C [10,11]. When dropping below this same temperature threshold (30-32^0^C), shivering stops typically allowing for core temperature to fall further at a faster rate. Achieving this threshold temperature rapidly with active warming (external or internal) will then allow the patients to contribute to their core warming by shivering [12].

## Conclusion

Despite the potentially high mortality for hypothermia with DKA, it may be worthwhile continuing aggressive resuscitation efforts even for prolonged periods before attaining ROSC for OHCA in patients found hypothermic from metabolic illnesses as compared to only from environmental exposures (avalanche victims, cold-water submersions, etc.) as has been traditionally reported in the medical literature. The foundation of this recommendation is the theory that DKA patients may have slowly achieved a core temperature below 25^0^C due their systemic illness prior to cardiac arrest, so that neuroprotection occurs prior to their no-flow state. The inverse situation of the cardiac arrest occurring while the temperature is still falling during avalanche asphyxiation or cold-water drowning portends less favorable neurologic outcomes.

[Since this manuscript does not contain any identifying patient information it was deemed to not require signed informed consent by our Institutional Review Board.]
